# Self-directed learning by video as a means to improve technical skills in surgery residents: a randomized controlled trial

**DOI:** 10.1186/s12909-021-02524-y

**Published:** 2021-02-05

**Authors:** Geneviève Chartrand, Mikael Soucisse, Pierre Dubé, Jean-Sébastien Trépanier, Pierre Drolet, Lucas Sideris

**Affiliations:** 1 Department of Surgery, Hôpital Maisonneuve-Rosemont, Université de Montréal, 5415 Boulevard l’Assomption, Montréal, Québec H1T 2M4 Canada; 2 Centre d’acquisition des attitudes et habiletés cliniques, Université de Montréal, 5415 Boulevard l’Assomption, Montréal, Québec H1T 2M4 Canada

**Keywords:** Surgery, Video, Residency, Pedagogy, Self-directed learning

## Abstract

**Background:**

With their demanding schedules, surgical residents have limited time to practice techniques. The aim is to evaluate the pedagogic model of self-directed learning using video in surgery residents.

**Methods:**

Informed consent was obtained from all the participants. A randomized controlled trial was conducted in 2018 at Hôpital Maisonneuve-Rosemont (University of Montreal). Participants were general surgery residents. There were 27 eligible residents; 22 completed the study. They were filmed performing an intestinal anastomosis on cadaveric pig bowel. The self-directed learning by video (SDL-V) group was given an expert video, which demonstrated the technique performed by an experienced surgeon. The control group continued with their regular duties. Three weeks later, participants performed a second filmed anastomosis. Two attending surgeons evaluated the residents’ filmed anastomosis using the Objective Structured Assessment of Technical Skills scale. After their second anastomosis, all participants had access to the expert video and completed a survey.

**Results:**

Score did not differ significantly between groups during the first (control: 23.6 (4.5) vs. SDL-V: 23.9 (4.5), *p* = 0.99, presented as mean (SD)) or second filmed anastomosis procedure (control: 27.1 (3.9) vs. SDL-V: 29.6 (3.4) *p* = 0.28). Both groups improved significantly from pre- to post-intervention (mean difference between the two anastomosis procedure with 95% CI for control: 3.5, [1.1; 5.9] and for SDL-V: 5.8, [3.4: 8.2]). Correlation between the evaluators for score was moderate (r = 0.6, 95% CI: [0.3: 0.8]). The pass/fail global evaluation exhibited poor inter-rater reliability (Kappa: 0.105, 95% CI: [− 0.2:0.4]). On the survey, all participants wanted more expert-made videos of specific surgical techniques.

**Conclusions:**

Despite a higher final OSATS score for the intervention group, self-directed learning by video failed to produce a statistically significant difference on the overall OSATS scores between the two groups in this small cohort.

**Supplementary Information:**

The online version contains supplementary material available at 10.1186/s12909-021-02524-y.

## Background

Throughout their surgical training, residents must acquire both theoretical knowledge and technical skills. These skills derive from the psychomotor development of residents, which links cognition to movement [1]. Despite work hour restrictions, residents’ schedules remain extremely busy with both hospital and academic demands. This time-constraint schedule leaves limited time to practice surgical techniques. Thus, it is imperative to investigate optimal teaching methods for surgical residents.

Simulation is widely used in surgical residency programs. Notable examples include the “Fundamentals of Laparoscopic Surgery” (FLS) course [2], which evaluates basic laparoscopic competencies on trainer boxes, and institutions with laboratories that use animal models to learn and practice surgical procedures [3]. There are many known benefits of teaching through simulation, such as improved skill acquisition and increased safety in patient care [4, 5].

More recently, studies have investigated the use of video coaching in surgical training. During a coaching session, the novice will receive directed feedback from an expert in the specific field. These studies have shown improved technical skills in intestinal anastomosis on canine models [6] and also in the operating room [7].

Auto-didacticism refers to the process by which the students organize their thoughts, behaviors and emotions to maximally reap rewards from a learning experience [8]. The learning benefit stems from the students’ motivation and autonomy. In this article, auto-regulation, auto-didacticism, and self-learning will be used interchangeably.

Two studies from the United Kingdom have compared the effects of personalized feedback from an expert versus a video made by an expert highlighting key points [9, 10]. In both cases, the medical students performed procedures (sutures, Foley catheter insertion, etc.) while being filmed. Students would then review their video with an expert or alone with a video made by an expert. The results showed that auto-didacticism was equally beneficial as the presence of an expert to improve the procedural skills. These studies, conducted with medical students and basic procedures, open the doors to a potential novel teaching method that should be investigated with surgical residents.

With the proliferation and accessibility of new technologies, a wide range of e-learning tools have developed to improve medical and surgical education [11]. These e-learning platforms span from online curricula to interactive modules. There are multiple advantages, such as availability of the material independent of the presence of an instructor. Studies in surgical disciplines have looked into e-learning interventions to improve knowledge acquisition for trainees and patient care. In gynecology, a blended learning program using e-learning videos with face-to-face training modalities reduced the incidence of obstetric anal sphincter injuries [12]. Another study gave surgical residents access to internet-based interactive modules [13]. Residents had increased course score after completing the module and the tool was recommended by most who participated. Another group created instructional videos detailing laparoscopic suture and knot tying [14]. While their findings did not demonstrate improved performance, they concluded that optimizing this method might lead to a useful teaching technique. A 2016 systematic review concluded that most surgical e-learning models are effective, but there remained significant heterogeneity in terms of application [15].

The goal of our study was to determine the efficacy of self-directed learning by video on the acquisition of a surgical procedure in surgical residents. The primary outcome was the impact of auto-didacticism on the Objective Structure Assessment of Technical Skills (OSATS) total score [16]. The secondary outcomes were the impact of auto-didacticism on the various sub-categories of the OSATS chart, the interrater reliability for OSATS score, the interrater correlation for Pass/Fail, and lastly, the results of the survey.

## Methods

### Design, setting and participants

This project was approved by the “Comité d’Évaluation Scientifique en Santé Physique” (Scientific Evaluation Committee) affiliated with Hôpital Maisonneuve Rosemont. Informed consent was obtained from all the participants. The inclusion criterion for participants in this project was being a surgical resident from the University of Montreal’s general surgery residency program, post-graduate year 1 (PGY-1) to PGY-5 who consented to participate. The exclusion criteria were not consenting or being unable to participate, due to scheduling issues, for instance. This study is a single blinded randomized controlled trial. There were two arms; a self-directed learning by video group (SDL-V) and a control group (C). The study took place in 2018 at the Surgical Simulation Laboratory at Hôpital Maisonneuve-Rosemont, a hospital affiliated with University of Montreal. All methods were carried out in accordance with relevant guidelines and regulations.

### Intervention

#### Baseline assessment

Residents were asked to create an end to end, single layer intestinal anastomosis with interrupted sutures on a cadaveric pig bowel. The same surgical assistant was present for all the procedures to ensure uniformity. Participants were told that they should inform this person exactly how they needed to be assisted during the anastomoses; otherwise the assistant did not take any initiative. This assistant also started and ended the recording, using a camera (*SONY Handycam Exmor R*) mounted on a tripod.

The intestines were harvested from pigs used in prior teaching modules (CPA 2016-NO-026, approved by Comité de Protection des Animaux du Centre de Recherche de l’Hôpital Maisonneuve-Rosemont). They were resected, divided into 15 cm long sections, and immediately frozen so that they could be thawed individually for each participant.

The Audiovisual Department of Maisonneuve-Rosemont Hospital edited the recordings. The final videos had no sound and repetitive tasks, such as knot tying, were removed.

#### Self-directed learning by video

After completing their baseline anastomosis, participants randomized to the intervention group received a Google Drive link (Mountain View, CA, USA) containing an expert video. This video was made prior to all baseline assessments. Producing the expert video took approximately 45 min. It demonstrates an end-to-end, single layer intestinal anastomosis using interrupted sutures on pig bowel. An experienced surgeon, who practices at the teaching hospital, performed the procedure. The video was finalized with voice-over narration by the same surgeon explaining key steps. The duration of this video was approximately 5 min. Residents were instructed to watch the video at their leisure and as many times as they wanted, they were also instructed not to share the video with other residents. Approximately 3 weeks after their baseline anastomosis, residents returned to the laboratory to perform the same procedure, which was also filmed.

#### Control group

After completing their baseline anastomosis, participants randomized to the control group did not have access to the expert video and were also asked not to view any videos online or elsewhere. They continued their regular clinical duties and returned to perform the same procedure, 3 weeks after their baseline anastomosis. After completion of their second anastomosis, they received the Google drive link, so they could also benefit from the expert video.

Residents, regardless of group, did not have access to the pig bowel between their baseline and second anastomosis to limit uneven skill acquisition through laboratory practice.

### Outcomes measured

All participants performed two anastomoses during two separate sessions approximately 3 weeks apart. All the anastomoses were performed on a 15 cm thawed pig bowel that had been cut in half by the same assistant. The interventions were filmed in the same manner, such that only the gloved hands of the participants, with their reference number written on them, could be seen. A video bank was created on a separate Google drive. The final videos had no sound.

The primary outcome was the total score of the Objective Structured Assessment of Technical Skills (OSATS) scale [16]. This operative performance rating scale contains the following seven categories: respect for tissues, time and motion, instrument handling, knowledge of instrument, flow of operation, use of assistants, and knowledge of specific procedure. Each category is rated from 1 (lowest) to 5 (highest). The maximum score is 35. The evaluator then decides if the resident passed or failed the intervention, this is determined by the holistic impression and not a cut-off score. Two experienced surgeons were given access to the video bank and scored each performance individually.

The OSATS scale has been validated for construct validity, internal consistency and inter-rater reliability for evaluating open surgical simulation procedures. Given that we had two evaluators, who independently reviewed all the videos, we also looked at the inter-rater consistency in our study.

Finally, two short surveys were created by the authors. They were sent to all the participants after they had completed the second filmed anastomosis; one was sent to the intervention group and the other to the control group. They were composed of questions with multiple choice answers (see additional file 1 for full surveys). They were designed to gauge the residents’ attitudes toward self-directed learning by video in the context of their surgical training.

#### Sample size

Given that the pool of possible participants were surgical residents at the University of Montreal, the study relied on a convenient sample. There were 27 eligible candidates, 5 of which were excluded (due to scheduling or “away rotation” reasons), thus 22 residents participated in the study.

#### Randomization

After making their baseline anastomoses, participants were randomly allocated to either the control group or the intervention group. This was done using a 1:1 ratio and block randomization, stratified by the residents’ post-graduate year. Block size varied according to the number of residents per year who consented to participate. A sealed envelope method was used for the randomization process. The two surgeons who scored the videos were blinded to the randomization.

### Statistical analysis

The PGYs of the participants of the two groups were compared with the Chi-square test. Spearman’s correlation coefficient was used to analyze the association between the OSATS scores (total and various components) provided by the two evaluators. Cohen’s Kappa coefficient was used to determine the inter-rater reliability between the two evaluators in terms of pass/fail. The intervals between the two sets of anastomoses of the groups were compared with the unpaired student-t test. Repeated measures two-way ANOVA followed with post-hoc Sidak’s multiple comparisons test was performed to compare the OSATS scores (total and various categories) of the two sets of anastomoses within and between each group. A *p* < 0.05 was considered significant. Statistical Analysis was done using PRISM 8.0 (Graphpad Software, La Jolla, CA). Since we used a convenient sample, we retrospectively calculated the size of the between-group difference in total OSATS scores that the experiment was powered to detect (statpages.info/postpowr.html). Unless stated otherwise, data are presented as mean (SD).

## Results

**Fig. 1:**
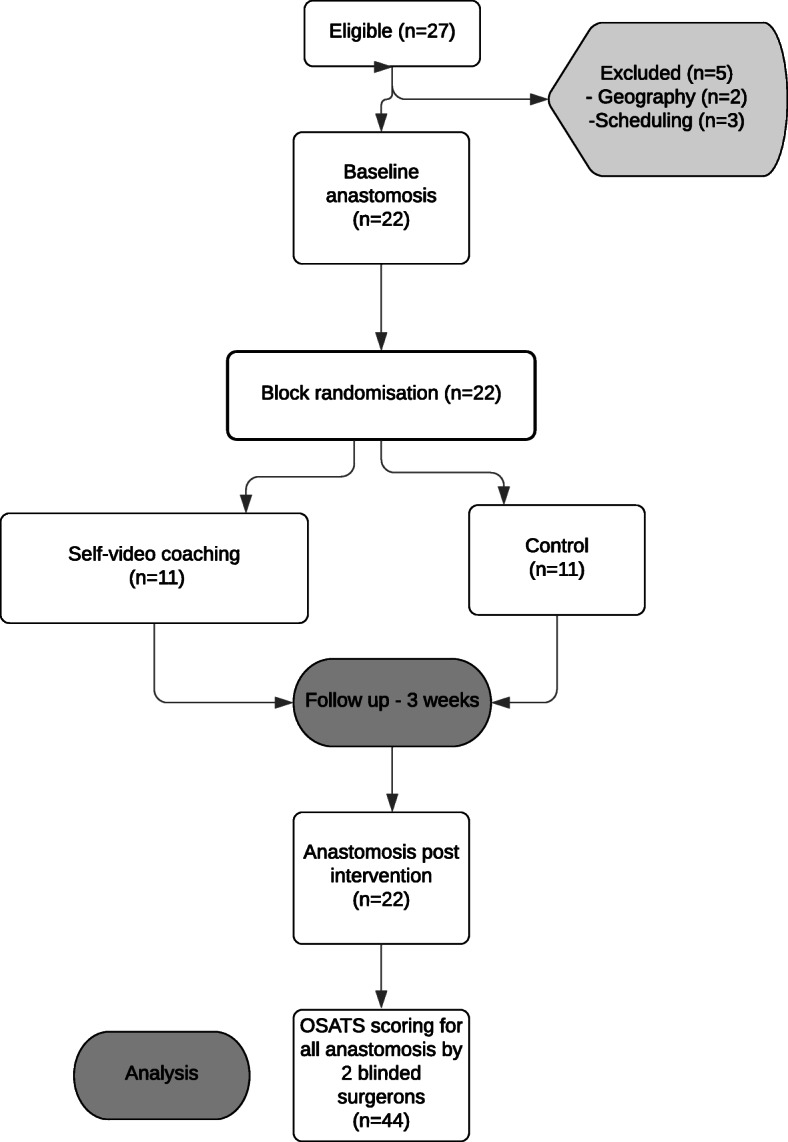
Study Flow Chart

There were 27 general surgery residents in our program. Two residents were unable to participate due to away rotations and 3 were unable to participate due to scheduling issues. Thus, 22 participants were consented for the study. There were 11 participants that completed the study per group (Fig. 1).

Baseline OSATS score and resident distribution per year were similar in both groups (Table 1).

**Table 1:** Baseline score, group distribution, and day interval between anastomosis. OSATS score and day interval presented as mean (SD). OSATS = objective structured assessment of technical skills. PGY = post graduate year. SDL-V = self-directed learning by video

	Control (***N*** = 11)	SDL-V (***N*** = 11)	***p***-value
Days between baseline and test anastomosis	26 (6)	27 (7)	*P* = 0.65
Baseline OSATS score (total)	23.6 (4.5)	23.9 (4.5)	*P* = 0.9
**Group allocation per PGY year**			
PGY1	2	3	–
PGY2	2	2	–
PGY 3	2	2	–
PGY 4	3	2	–
PGY 5	2	2	–

### OSATS results

**Fig. 2:**
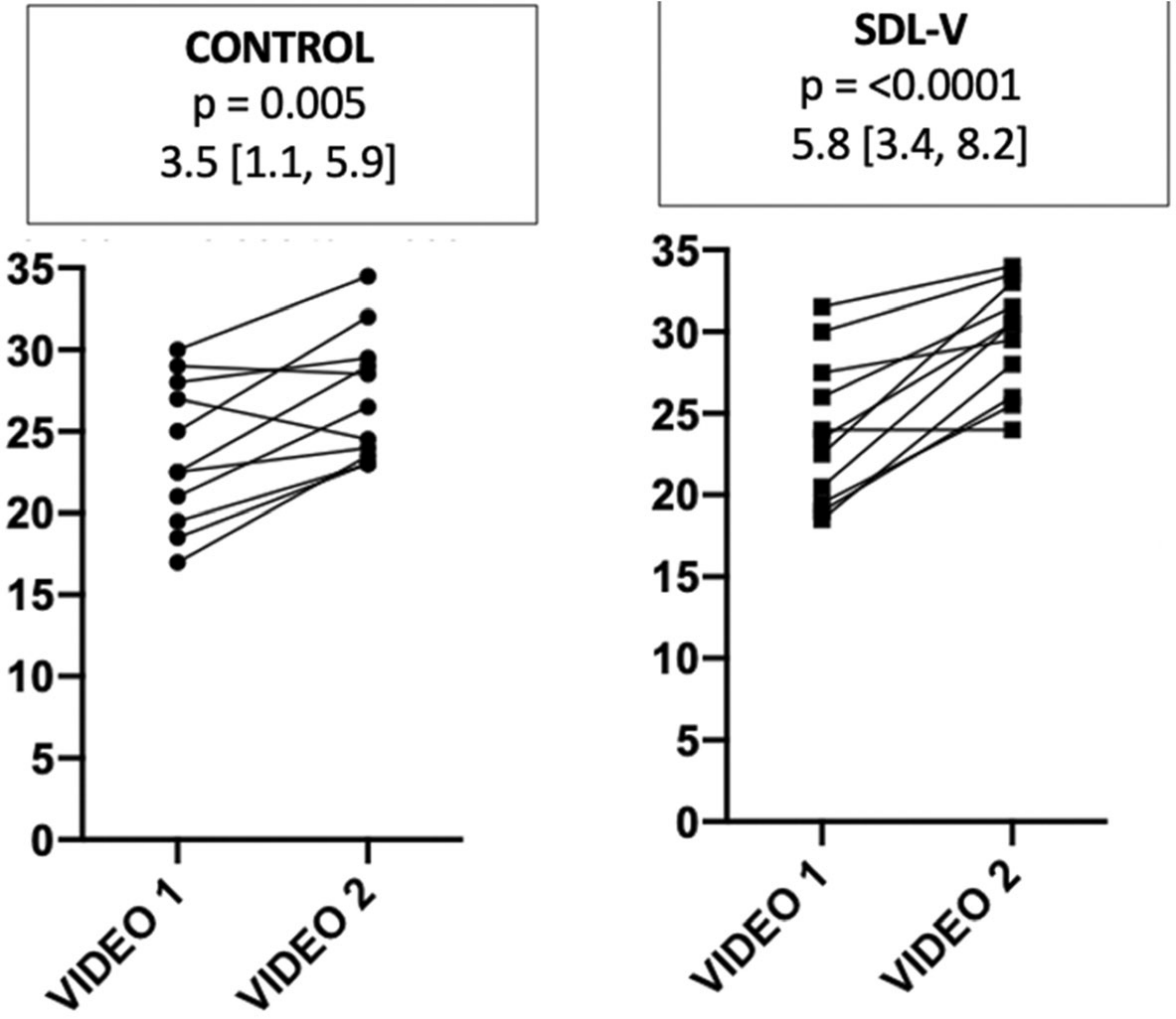
Mean difference in total OSATs score (presented as mean difference and 95% confidence interval) between first and second video for the control and intervention groups

There was no difference between groups regarding the total OSATS score during the initial (control: 23.6 (4.5) vs. SDL-V: 23.9 (4.5) *p* = 0.9) or the second filmed anastomosis procedure (control: 27.1 (3.9) vs. SDL-V: 29.6 (3.4), *p* = 0.28), but both groups significantly improved their performances during the interval (mean difference between video 1 and 2 with 95% CI in control: 3.5 [1.1; 5.9] and SDL-V: 5.8 [3.4; 8.2]) (Fig. 2.). Neither the groups (2.3%) nor the interaction between groups and videos (1.6%) were a significant source of variation during the RM ANOVA analysis of the total OSATS scores. Regarding the various OSATS components, both groups improved significantly in four of the seven categories (respect of tissue, time and motion, flow of operation and use of assistant) while only the SDL-V group improved in the three others (instrument handling, knowledge of instruments and knowledge of specific procedural steps) (Table 2). However, no difference was found between the groups for the first or the second filmed anastomosis procedure in any of the OSATS categories; in all categories, neither the groups nor the interaction between groups and videos were a significant source of variation during the RM ANOVA analysis.

**Table 2:** Mean score improvement (with 95% confidence interval) and *p*-value, per group, per OSATS category. CI = confidence interval. OSATS = objective structure assessment of technical skills

	Control Group		Intervention Group	
OSATS category	Mean [95% CI]	***P*** value	Mean [95% CI]	***P*** value
**Respect of tissues**	0.9 [0.2, 1.5]	*P* = 0.008	1.2 [0.5, 1.8]	*P* = 0.0005
**Time and Motion**	0.6 [0.1, 1.2]	*P* = 0.02	0.7 [0.2, 1.2]	*P* = 0.006
**Instrument Handling**	0.2 [−0.3, 0.7]	*P* = 0.5	0.5 [0.1, 1]	*P* = 0.03
**Knowledge of instruments**	0.1 [−0.5, 0.7]	*P* = 0.9	0.7 [0.1, 1.3]	*P* = 0.02
**Flow of operation**	0.5 [0.03, 1.1]	*P* = 0.03	1.1 [0.6, 1.6]	*P* < 0.0001
**Use of assistant**	0.5 [0.09, 0.8]	*P* = 0.01	0.7 [0.3, 1.04]	*P* = 0.0004
**Knowledge of procedure**	0.6 [−0.07, 1.3]	*P* = 0.08	5.7 [3.4, 8.1]	*P* = 0.001

### Retrospective power analysis

Having found a non-significant difference of 2.6 (*p* = 0.28) in total OSATS scores between the groups for the second video, a retrospective sample size analysis showed that 36 participants would have been necessary to deem a variation of such magnitude as significant. With the 11 participants that were used, power was sufficient to detect a mean difference of 4.61.

### Inter-rater reliability

Two attending surgeons at our institution independently evaluated each filmed anastomosis, and were blinded to the study group. There was a statistically significant correlation between the overall score (the tally of each OSATS category) between the two evaluators, *r* = 0.58 (95% CI 0.34–0.75, *P* < 0.0001). OSATS score for each category were also significantly correlated, with the singular exception for “knowledge of instruments” (Table 3). However, for the pass/fail results, the evaluators agreed for 68% of the videos. Since the number of agreements expected by chance was 64%, resulting in a Cohen’s Kappa coefficient value of 0.105 (95% CI: − 0.20 to 0.41), the correlation was considered “poor.” The percentage of participants deemed to have passed or failed per filmed anastomosis per group for each evaluator is detailed in Table 4.

**Table 3:** Correlation between evaluators on OSATS score. OSATS = objective structured assessment of technical skills

Category	R	95% CI	***P*** value
Respect of tissue	0.41	0.12 to 0.64	0.0053
Time and motion	0.46	0.18 to 0.67	0.0019
Instrument handling	0.38	0.08 to 0.61	0.012
Knowledge of instruments	0.11	−0.20 to 0.39	0.49
Flow of operation	0.54	0.27 to 0.72	0.0002
Use of assistant	0.33	0.02 to 0.57	0.031
Knowledge of procedural steps	0.5	0.23 to 0.69	0.0006

**Table 4:** Percentage of participants (n in parenthesis) deemed to have passed, per first and second filmed anastomosis, for each rater

	First filmed anastomosis		Second filmed anastomosis	
	Control	Intervention	Control	Intervention
Rater 1	55 (6/11)	55 (6/11)	82 (9/11)	100 (11/11)
Rater 2	64 (7/11)	64 (7/11)	91 (10/11)	91 (10/11)

### Survey

In the control group, 2 candidates reported watching a video on intestinal anastomosis and performing one during a real-case procedure. Every candidate in the control group wished they had had access to the video.

In the intervention group, all candidates watched the video 1 to 2 times between the two anastomoses. This self-teach didactic activity was considered enjoyable and useful by all.

Every candidate, regardless of group, reported wishing they had access to similar videos on various techniques throughout their residency.

## Discussion

General surgery residency is a 5-year training program. During this time, the residents’ role is divided between being a healthcare provider and learner. In North America, residents can work between 70 to 100 h per week, depending on the specialty [17, 18]. Surgical residency is notorious for its long hours. Furthermore, the knowledge acquired in a surgical program must be both cognitive and technical. Thus, with limited time, the learning experience requires optimization. The traditional Halsteadian method of teacher-apprentice can no longer provide residents with sufficient operative experience and is often simply not feasible [19–21].

Our focus was to investigate a practical, focused learning method that was amenable to the residents’ schedule. E-learning encompasses a wide array of learning techniques, from online reading to interactive multimedia platforms. Its use to optimize medical pedagogy is under investigation as it has several advantages (accessible, can contain extensive material, interactive, independent of instructor availability) [22, 23]. Studies have tried to assess its use as a stand-alone method and in conjunction with other learning techniques.

In this study, we designed our e-learning technique. We hypothesized that creating a short video, tailored specifically for a single technique, voiced over by an expert in the field, could provide a useful tool for the residents. The use of this method would give the residents flexibility in their learning; it could quickly and easily be viewed at any time, even on the ubiquitous smart phone. The material was made by a reliable source, and the narration provided key points. The residents universally appreciated the experience and all the candidates reported wanting more similar videos.

The intervention group had a statistically significant improvement in all individual OSAT categories between their first and second filmed anastomosis, compared to the control group, which improved in only 4 of the 7 categories. Perhaps the intervention is a useful tool to hone those specific skills (instrument handling, knowledge of instruments and knowledge of specific procedural steps). This could be evaluated more closely in a follow up study.

However, despite a higher final OSATS score for the intervention group, there was no statistically significant difference between those who had access to the video and those who did not. Since both groups improved, it is plausible that simply performing two anastomosis a few weeks apart was sufficient to improve the skill. This could also be due to the effect of mental imagery or mental rehearsal; that despite not having access to the expert video, the control group still improved on the skill [24]. On the survey, two candidates in the control group reported seeing a video of the procedure, which can also explain the improvement. The diffusion of our expert video from the intervention group to the control group seems highly unlikely, though cannot be entirely ruled out.

There are some limitations to our study. It is possible that the lack of significance is due to a small number of participants: there were 27 residents in our program, and 22 were randomized. The retrospective power analysis indicated a minimum of 18 participants per group to show a difference. Also, subtle technical differences may be lost in the quality of the filming, which was done with a stationary camera. The procedural task may have been too simple to show a significant difference. Finally, the intervention itself could have no impact in terms of acquisition of technical surgical skill.

In terms of the inter-rater reliability, studies have previously shown a correlation coefficient for OSATS scores close to 0.8 [16]. In our study, the correlation coefficient was moderate, at 0.58. The different result from our study compared to the literature could possibly due to our study’s limitations. In several pedagogic studies, despite a correlation for scaled items, holistic views on whether or not a candidate passes or fails tend to have poor agreements between evaluators [25, 26]. Therefore, though our kappa score for the pass/fail results is poor this may not be that surprising.

Given the positive response to this intervention, there may be a place for similar learning techniques in the surgical curriculum. With patient consent, real interventions (such as appendectomies, cholecystectomies, bowel resections) could be filmed, edited and narrated. A video library could be created for residents. This could be coupled with current technical workshops and lectures that several surgical programs usually offer.

## Conclusion

In our study, despite a global positive appreciation of the experience, self-directed learning by video did not prove to be a significant learning tool. It is conceivable that the number of participants was insufficient. With the widespread use of technology, there is certainly a role for video or e-learning in the surgical curriculum [27], and further research in this subject is pertinent.

## Supplementary Information

**Additional file 1.**

## Data Availability

The datasets generated and analysed during the current study are restricted to protect participant anonymity. Information may be available from the corresponding author on reasonable request.

## Abbreviations

SDL-V: self-directed learning by video
FLS: Fundamentals of Laparoscopic Surgery
OSATS: Objective Structure Assessment of Technical Skills
PGY: post-graduate year
