# Supplementary material for: Self-directed learning by video as a means to improve technical skills in surgery residents: a randomized controlled trial
Source: BMC Med Educ. 2021 Feb 5;21:91. doi: 10.1186/s12909-021-02524-y (PMC7863545; doi:10.1186/s12909-021-02524-y)
Supplement: Supplementary file 1 — Additional file 1. [file 12909_2021_2524_MOESM1_ESM.pdf]

Additional file 1

SELF-DIRECTED LEARNING BY VIDEO AS A MEANS TO IMPROVE TECHNICAL SKILLS IN SURGERY RESIDENTS: A RANDOMIZED CONTROLLED TRIAL.

Geneviève Chartrand, Mikael Soucisse, Pierre Dubé, Jean-Sébastien Trépanier, Pierre Drolet, Lucas Sideris MD.

Survey 1 and 2 sent to the intervention and control groups, respectively, after the second filmed anastomosis. Surveys were web-based and anonymous.
(Surveys translated from French.)

Survey 1 (intervention group)

Question	Answer options
How many times did you watch the video?	a. 0<br>b. 1<br>c. 2<br>d. >3
Is this self-directed learning technique enjoyable?	a. Yes<br>b. Neutral<br>c. No
Is this self-directed learning technique useful?	a. Yes<br>b. Neutral<br>c. No
Would you like to have access to similar videos during your residency?	a. Yes<br>b. Neutral<br>c. No
The duration of the video was	a. Too short<br>b. Perfect<br>c. Too long

Survey 2 (control group)

Question	Answer options
Did you watch a movie about the technique between the two anastomoses?	a. Yes<br>b. no
Between the two anastomoses, did you perform this technique in the operating room?	a. Yes<br>b. no
Would you have wanted to see the video?	a. Yes<br>b. No<br>c. I saw another video
Would you like to have access to videos about techniques during your residency?	a. Yes<br>b. Neutral<br>c. No
